# Prevention of Cardiovascular Disease: Screening for Magnesium Deficiency

**DOI:** 10.1155/2019/4874921

**Published:** 2019-05-02

**Authors:** Paolo Severino, Lucrezia Netti, Marco Valerio Mariani, Annalisa Maraone, Andrea D'Amato, Rossana Scarpati, Fabio Infusino, Mariateresa Pucci, Carlo Lavalle, Viviana Maestrini, Massimo Mancone, Francesco Fedele

**Affiliations:** ^1^Department of Cardiovascular, Respiratory, Nephrology, Anesthesiology and Geriatric Sciences, Sapienza University of Rome, Viale del Policlinico 155, 00161 Rome, Italy; ^2^Department of Human Neurosciences, Sapienza University of Rome, Viale dell'Università 30, 00185 Rome, Italy

## Abstract

Magnesium is an essential mineral naturally present in the human body, where it acts as cofactor in several enzymatic reactions. Magnesium is a key cardiovascular regulator, which maintains electrical, metabolic, and vascular homeostasis. Moreover, magnesium participates in inflammation and oxidative processes. In fact, magnesium deficiency is involved in the pathophysiology of arterial hypertension, diabetes mellitus, dyslipidemia, metabolic syndrome, endothelial dysfunction, coronary artery disease, cardiac arrhythmias, and sudden cardiac death. In consideration of the great public-health impact of cardiovascular disease, the recognition of the negative effects of magnesium deficiency suggests the possible role of hypomagnesaemia as cardiovascular risk factor and the use of serum magnesium level for the screening and prevention of cardiovascular risk factors and cardiovascular diseases. Moreover, it might help with the identification of new therapeutical strategies for the management of cardiovascular disease through magnesium supplementation.

## 1. Introduction

Magnesium (Mg^2+^) is an essential mineral naturally present in the human body, where it plays an important role as a cofactor in about 325 enzymatic reactions such as the production of adenosine triphosphate (ATP), the synthesis of nucleotides, glucose, and blood pressure control, and lipid peroxidation [1]. Mg^2+^ is the second most important intracellular cation after potassium (K^+^) and is fundamental in muscle contraction, nerve conduction, and bone strength. In an adult human body, there are approximately 0.4 g Mg^2+^/kg [2], with about 50–60% localized in bones, and the rest distributed in skeletal muscle and soft tissues. Serum Mg^2+^ represents a little percentage, less than 1% of all Mg^2+^ in the body [3], and the normal reference range is 0.76–1.15 mmol/L [4]. However, bone Mg^2+^ is largely exchangeable to counteract acute changes in serum levels of this mineral, while one-third of skeletal Mg^2+^ accomplishes the same function [5]. We can obtain Mg^2+^ from different types of food as green leafy vegetables, legumes, cereal and nuts, which have a great Mg^2+^ content, or fruits, meat, fish, and chocolate, providing a moderate amount of Mg^2+^. Moreover, water represents an important source of Mg^2+^, because it contains up to 30 mg/L of Mg^2+^ [6].

Scientific literature has reported the role of Mg^2+^ as important cardiovascular regulator, acting to maintain electrical, metabolic, and vascular homeostasis; additionally, Mg^2+^ modulates inflammation and oxidative processes that are known to be triggers for atherogenesis and cardiovascular diseases (CVDs) [7]. More recently, several data have shown the association between Mg^2+^ intake and circulating Mg^2+^ with CV health [7–9]; hypomagnesaemia has been associated to increased risk of type 2 diabetes mellitus (T2D), metabolic syndrome (MetS), arterial hypertension, endothelial dysfunction, and CVD. Hence, new evidences suggest that hypomagnesaemia may have a detrimental effect on CV health and may increase the total risk of developing several metabolic conditions and CVD. Dietary surveys have shown deficient Mg^2+^ intake in a large proportion of population, probably due to Western dietary habits. Additionally, other known causes of hypomagnesaemia, such as intestinal malabsorption, gastrointestinal losses, and diuretics or lassative assumption, are very frequent in general population but often underestimated and undertreated, particularly among elderly individuals.

The recognition of the possible role of hypomagnesaemia as risk factor for CV health, along with the underestimation of the importance of this mineral in daily clinical practice, makes serum Mg^2+^ level suitable for the screening and prevention of CVD and opens new therapeutic scenarios with the possibility of reducing CV risk profile and treating CVDs through Mg^2+^ supplementation.

## 2. Mg^2+^ and Cardiovascular System: Pathophysiologic Insights

Mg^2+^ exerts beneficial effects on the cardiovascular system by acting on transmembrane ion transport pumps, improving glucose and insulin metabolism, enhancing endothelium-dependent vasodilation, improving lipid profile, and acting as an antihypertensive and anti-inflammatory agent [10]. Additionally, Mg^2+^ is a natural calcium antagonist, is an essential cofactor in mitochondrial oxidative reactions, and has anticoagulant and antiplatelet properties.

### 2.1. Magnesium as Ionic Channel Regulator

Mg^2+^ participates to the control of the activity of some ionic channels, such as sodium (Na^+^), potassium (K^+^), and calcium (Ca^2+^) [11, 12]. Mg^2+^ reduces the rapid inward component of the delayed rectifier K^+^ channel (I_Kr_) [13] and exerts an antiarrhythmic action modulating the duration of action potential and myocardial excitability. In fact, Mg^2+^ infusion provokes the slowing of atrioventricular (AV) nodal conduction and also determines the prolongation of PR and QRS duration [14]. Mg^2+^ prolongs both atrial and ventricular refractory period, reducing proarrhythmic substrate for triggered automaticity and reentry circuits [15, 16]. On the contrary, Mg^2+^ deficiency is correlated with a prolonged QT interval potentially associated to the development of ventricular arrhythmias as the torsade de pointes. Magnesium sulfate (MgSO_4_) is usually successfully used during the episodes of torsade de pointes, because it is able to stop early after depolarizations (EADs) and automaticity by decreasing I_Kr_ current and blocking long-lasting type (L-type) Ca^2+^ activity [17]. Through the inhibitory effect on two Ca^2+^ channels, transient-type (T-type) and L-type [18], Mg^2+^ exerts a protective effect against triggered activity, prevents coronary artery spasm, and plays a crucial role in modulating vascular muscle tone and, therefore, systemic arterial blood pressure. Furthermore, Mg^2+^ plays a critical role in the potassium-proton exchange and in the protection against potassium loss. Hypomagnesaemia weakens this mechanism and also promotes the augmentation of intracellular Na^+^ and Ca^2+^ concentrations. These concepts can explain how hypomagnesaemia can impact the physiological activity of muscle cells in general and cardiomyocytes and vascular muscle cells in specific, leading to cardiovascular diseases and coronary vasospasm [19]. Additionally, myocardial ischemia is related to intracellular Ca^2+^ overload that has detrimental effect on myocardial function. Competing for the same binding sites, Mg^2+^ may reduce intracellular Ca^2+^ overload during myocardial ischemia, and may restrict infarct size limiting coronary artery spasm, reducing postinfarction oxidative damage [20], and improving endothelial-dependent vasodilation through NO release [21].

### 2.2. Magnesium as Enzymatic Cofactor

Mg^2+^ acts as a cofactor in multiple cellular reactions, some of which take place in mitochondria, that also represent the main intracellular reservoir of this ion and, through the Mg^2+^ transporter, mediate the redistribution of Mg^2+^ inside cells according to physiological stimuli [22]. Dysregulation in homeostasis of mitochondrial Mg^2+^ has effects both on mitochondrial energy production and mitochondrial morphology, disrupting ATP production [22].

### 2.3. Magnesium and Metabolic Homeostasis

Mg^2+^ seems to have beneficial effects on CV health, reducing the risk of developing type 2 diabetes mellitus and metabolic syndrome [23, 24]. This ion improves glucose and insulin metabolism modulating the activity of glucose transporter protein 4 (GLUT 4) [25] and enhancing insulin sensibility. Particularly, it has been demonstrated that Mg^2+^ mediates pancreatic insulin release, postreceptor insulin signaling, and acts as a second messenger for insulin-mediated signal transduction [26, 27].

### 2.4. Magnesium and Inflammatory Response

Mg^2+^ has been associated to the regulation of inflammation and oxidative processes. Indeed, this ion has anti-inflammatory effect, modulating the release of nuclear factor-kB [28], and antioxidant properties, by scavenging oxygen radicals [28–34]. The inflammatory response that takes place in case of hypomagnesaemia also influences the lipid profile through lipoprotein peroxidation [35], with the increase of the triglyceride-rich lipoproteins, the accumulation of plasma concentrations of apolipoprotein B, decreased high-density lipoprotein (HDL) levels resulting in the development of dyslipidemia [34]. These lipoprotein changes, together with the chronic impairment of redox status, lead to the atherogenic effects of hypomagnesaemia and exalt the possible role of Mg^2+^ in the cardiovascular health.

### 2.5. Magnesium and Coagulation System

Mg^2+^ has anticoagulant [36] and antiplatelet properties. The latter is based on the Mg^2+^ capacity to reduce platelet activation, both by inhibiting the production of platelet stimulating factors, such as thromboxane A2 (TXA2), and *e* increasing the release of platelet inhibiting factors, such as prostacyclin (PGI2) [37, 38], and to counteract platelet aggregation, because it competes with Ca^2+^ ions for specific sites in the glycoprotein (Gp) IIb subunit, modifying the receptor conformation and hindering fibrinogen-Gp IIb-IIIa interaction. This effect could be observed greatly in case of Mg^2+^ supplementation, as highlighted by Gawaz et al. [39]. Moreover, Mg^2+^ is able to inhibit thrombin-induced Ca^2+^ influx [40].

### 2.6. Magnesium and Microvascular System

Of note, hypomagnesaemia could increase the susceptibility to CVD affecting the endothelial function and modulating microvascular functions. The role of coronary microcirculation and its endothelial or nonendothelial-dependent dysfunction in the pathogenesis of CVD, and specifically of myocardial ischemia, is well known. Recent evidences in this field assigned a new visibility and importance to the presence of single-nucleotide polymorphisms (SNPs) in genes encoding for coronary ion channels, most of all for the ATP-dependent K^+^ channel (K-ATP), as a possible substrate where endothelial dysfunction establishes itself. Starting from these considerations, it is more intelligible how Mg^2+^, as essential intracellular cation, can affect these mechanisms [41–43]. Indeed, Mg^2+^ prevents vasospasm and endothelial damage [44] by stimulating endothelial proliferation and angiogenesis, upregulating the endothelial nitric oxide synthase (eNOS), thus increasing nitric oxide (NO) release. Conversely, low Mg^2+^ levels affect and reduce the synthesis of vasodilator molecules and slow the proliferation of endothelial cells and promote the adhesion of monocytes, increasing the susceptibility of the CV system to oxidative stress and contributing to the creation of a prothrombotic and atherogenic surface [26, 27, 33–35, 44–59]. The production of nitric oxide (NO) represents the main protective action performed by the coronary endothelium to avoid vasospasm or thrombosis and provides the endothelium-dependent vasodilation acetylcholine-induced. Pearson et al. demonstrated that Mg^2+^, infused after cardiac operations, affects endothelial-dependent vasodilation without interfere with NO production, and low extracellular Mg^2+^ levels affect NO-dependent vasodilation [60]. By reducing NO release and affecting Ca^2+^ handling system, Mg^2+^ deficiency modulates vascular tone and can increase the risk of developing hypertension [59, 61, 62]. It has been demonstrated that Mg^2+^ induces endothelial dysfunction reducing endothelial proliferation [63]. Particularly, the slowdown in endothelial cells proliferation is linked to the upregulation of interleukin-1 (IL-1), a potent inhibitor of endothelial growth [64], and, on the other hand, the downregulation of cell division cycle 25B (CDC25B) phosphatase with a role in the promotion of cells G2–M progression in cellular cycle [63, 65]. Furthermore, hypomagnesaemia results in the overexpression of adhesion molecules, like soluble vascular cell adhesion molecule 1 (sVCAM-1) and E-selectin, associated to the proinflammatory status with the increased release of interleukin-1a (IL-1) and interleukin-6 (IL-6) [52]. The upregulation of these endothelial-related markers results in an amplificated monocyte adhesion to the endothelium and promotes atherogenesis and CVD development [66].

## 3. Mg^2+^ and Cardiovascular System: Clinical Insights

**Figure 1:**
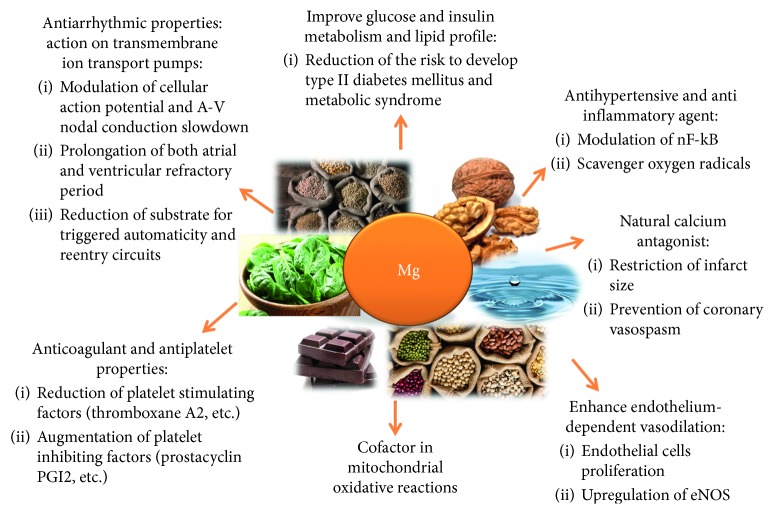
Molecular effects and role of Mg^2+^ in the cardiovascular system.

As mentioned above, Mg^2+^ is a fundamental mineral in cardiac health, and its role is essential in various different cardiovascular and metabolic conditions. It is a defense against the oxidative damage [67–69], a physiological Ca^2+^ antagonist [18] that contributes to the modifications in the membrane potential [70], a regulator of platelet aggregation and adhesion [37, 38], and a modulator of endothelial function [60, 63, 64]. There are several studies reporting an inverse association between serum Mg^2+^ level, Mg^2+^ supplementation, and CVD. These evidences suggest that this ion could be monitored for screening and prevention of CVD and possibly supplemented as an adjunctive pharmacotherapy for CVD (Figure 1).

Prevention and treatment of cardiac arrhythmias are the most widely accepted indications for Mg^2+^ use in daily clinical practice, since Mg^2+^ deficiency can disrupt the homeostasis of intra- and extracellular ions leading to the prolongation of QT segment, ST-depression, and T waves characterized by a lower amplitude [71]. Salaminia et al. conducted a meta-analysis of twenty-two randomized trials that highlighted the role of MgSO_4_ in the reduction of cardiac arrhythmias, showing the lowering of the risk of ventricular arrhythmias about 32% and supraventricular arrhythmias 42% [72]. This effect was confirmed also by other meta-analyses [73–75]. This suggested that the administration of MgSO_4_ could be a safe, effective, and cost-effective strategy for the preservation of cardiac patient's health [72]. The importance of Mg^2+^ in the chapter of arrhythmias is greater in the prevention against postoperative atrial fibrillation arising after coronary artery bypass [75] or after cardiac surgery [76, 77]. In the same way, the Framingham Heart Study highlighted how in the comparison between individuals belonging to the lowest quartile or the upper quartiles of serum Mg^2+^, the first group was about 50% more likely to go to atrial fibrillation than the latter. Therefore, it resulted that low-serum Mg^2+^ levels are moderately associated with the onset of atrial fibrillation also in people without cardiovascular diseases [78]. The risk of the development of ventricular arrhythmias is influenced by Mg^2+^ concentrations also. In addition to the well-known answer of torsade de pointes episodes to MgSO_4_, Raghu et al. demonstrated the efficacy of the Mg^2+^ supplementation, adjuvant to thrombolytic therapy, in preventing ventricular arrhythmias and reducing short-term mortality after acute myocardial infarction [79]. As a result, the European Society of Cardiology incorporated Mg^2+^ in their guidelines regarding the prevention and the managing of some kind of cardiac arrhythmias [80].

Mg^2+^ deficiency, through abnormalities of Ca^2+^ handling system, dysregulation of vascular tone and endothelial dysfunction, contributes to induce hypertension. A recent meta-analysis showed a statistically significant inverse association between Mg^2+^ intake and arterial hypertension [23]. These results were consistent with those by Dibaba et al. that pointed out the reduction of both systolic and diastolic blood pressure through Mg^2+^ supplementation [81].

It has been demonstrated that hypomagnesaemia alters the normal lipid profile and induces insulin resistance (IR), T2D, and MetS. A recent meta-analysis demonstrated a linear dose-response relationship between Mg^2+^ intake and T2D: the higher the Mg^2+^ intake, the lower the risk of T2D [23]; Sarrafzadegan et al. showed that Mg^2+^ supplementation was associated with lower risk of MetS [82]. Through oxidative stress and lipid peroxidation, Mg^2+^ deficiency has been linked to dyslipidemia, and in some meta-analysis, Mg^2+^ supplementation showed beneficial effects reducing triglycerides and total cholesterol [83, 84].

The association of Mg^2+^ deficiency with CV risk factors promoting the generation of atherosclerotic plaques is probably the reason of the increased risk of ischemic heart disease (IHD) seen in patients with hypomagnesaemia. Particularly, the risk of myocardial infarction is also referable to the role that Mg^2+^ plays in favoring oxidative stress, suggested by a lot of evidences both in humans and animal models [67]. Hans et al. studied in rats with Mg^2+^ deficiency the lowering of plasma anticoagulants and an increased lipid peroxidation [68], while Kuzniar et al. observed in mice that hypomagnesaemia led to the reduction of glutathione (GSH) levels and to a decrease of GSH reductase, superoxide dismutase, and GSH S-transferase activity in erythrocytes [69]. Kharb and Singh tried to understand if Mg^2+^ deficiency may promote oxidative injury in cardiovascular disease states and highlighted how actually low Mg^2+^ concentrations can accelerate and potentiate oxidative insult in the myocardium [85]. The link between hypomagnesaemia and IHD has been pointed out by a recent systematic review, showing that higher circulating Mg^2+^ levels are associated with lower risk of IHD and fatal IHD [9].

Another mechanism through which low Mg^2+^ levels can bring to IHD is coronary vasospasm, and this event is mostly due to the consequences of hypomagnesaemia on intracellular Ca^2+^. A lot of studies have already demonstrated that Mg^2+^ deficiency is correlated to coronary artery spasm [86–90]. Guo et al., who investigated the Mg^2+^ status in and out of cells in 12 women affected by variant angina, found that Mg^2+^ concentration in the cytoplasm of erythrocytes was well related to the activity of the variant angina [86]. Starting from this knowledge, Teragawa et al. studied whether Mg^2+^ could be useful in the prevention of coronary vasospasm in patients with vasospastic angina. They demonstrated that Mg^2+^ infusion can induce nonsite specific basal coronary vasodilation and block coronary spasm acetylcholine-induced in patients affected by vasospastic angina [87].

Recently, Joris et al. conducted a randomized, double-blind, and placebo-controlled intervention trial in a group of overweight and obese adults to study the effects of Mg^2+^ on arterial stiffness, a known marker of CV health. They found that Mg supplementation reduced the arterial stiffness without any effects on blood pressure values, suggesting a potential way through which an increased Mg^2+^ intake could exert benefits for cardiovascular health [91, 92].

A systematic review and meta-analysis conducted by Del Gobbo et al. tried to draw a link between circulating and dietary Mg^2+^ and risk of cardiovascular disease, such as ischemic heart disease, and it was confirmed with significant results, supporting recommendations to increase Mg^2+^ intake, mostly the consumption of Mg^2+^-rich food, as vegetables and nuts [9].

New evidences suggest that Mg^2+^ supplementation could have beneficial effects in the early phase of acute coronary syndrome and after implantation of drug-eluting stent (DES). Gyamlani et al. found that Mg^2+^ levels during and after acute myocardium infarction (AMI) were lower in patients affected by AMI than controls and that intravenous Mg^2+^ administered in the immediate postinfarction period in the patients had a cardioprotective effect and the capacity to reduce arrhythmias, pump dysfunction, and death [93]. A study conducted by An et al. showed that Mg^2+^ levels could be considered, independently from other risk factors, as predictor of major adverse cardiac events (MACEs) in patients who undergo a DES implantation after AMI, but not unstable angina [94]. Similarly, Yuksel et al. identified low Mg^2+^ as independent predictor of electrocardiographic no-reflow and long-term mortality in 111 patients after ST-segment elevation myocardial infarction (STEMI) and primary percutaneous coronary implantation (p-PCI) [95]. In continuity with these results, Çiçek et al. analyzed the hypothetical association between Mg^2+^ levels and acute stent thrombosis after a p-PCI in people with STEMI. The results pointed out that Mg^2+^ was an independent predictor of acute stent thrombosis in these groups of patients [96]. Besides, since Mg^2+^ represents an important ion in the determination of membrane potential, dosing its plasma levels, together with calculation of the concentrations of the other main ions, could be useful in the identification of the membrane permittivity and conductivity, for the purpose of better understanding the mechanisms that are at the basis of the hemocompatibility of vascular stent materials [97].

Nevertheless, data regarding the protective role of Mg^2+^ in the reduction of mortality after acute myocardial infarction are not all concordant, and there are conflicting results, such as the MAGIC (Mg^2+^ in coronaries) trial that did not report any advantage of early Mg^2+^ administration in addition to the standard therapy in high-risk patients after STEMI, with no effects on 30-day mortality [98]. Vassalle et al. did not observe a significant role of low Mg^2+^ levels in predicting hard events (all causes of death and nonfatal myocardial infarction) in patients after AMI [99]. The results found in these studies are probably due to the different population features, pointing out that Mg^2+^ supplementation could exert the beneficial effect in low-risk patients with CVD or in prevention of CVD rather than in patients with high CV risk profiles.

Finally, a big number of studies published in the past decades links hypomagnesaemia to sudden cardiac death (SCD) because of different reasons, such as the major frequency of sudden death in areas where community water supplies have a little Mg^2+^ content or the lower Mg^2+^ concentrations in people who die of sudden death. A possible explanation of this correlation is referable to cardiac arrhythmias and coronary vasospasm, which are usually associated to Mg^2+^ deficiency. An important study related to this question was conducted by Peacock et al. in a cohort of more than fourteen thousand persons, distributed according to the value of their serum Mg^2+^. The results showed that people at the higher quartile of serum Mg^2+^ had a significantly lower risk of sudden death compared to individuals at the lower quartile, and these findings were confirmed also after the adjustment for the major potential confounders of Mg^2+^-SCD relationship, like hypertension, diabetes, serum K^+^ concentrations, or use of diuretics [100].

All these evidences suggest that hypomagnesaemia may predispose to the development of CV risk factors and CVD. The awareness of the possible role of this ion as risk factor for CVD may lead to use Mg^2+^ level for screening and prevention of CVD and Mg^2+^ supplementation as adjunctive pharmacotherapy for patients suffering from heart disease.

**Figure 2:**
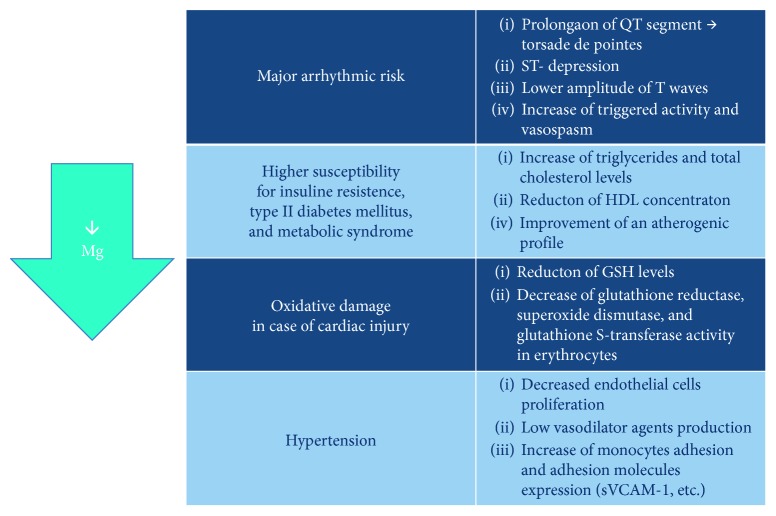
Detrimental consequences of Mg^2+^ deficiency on the cardiovascular health.

**Figure 3:**
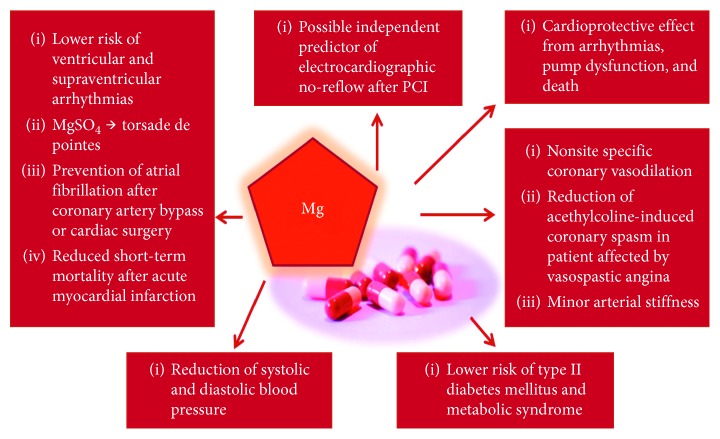
Potential benefits and role of Mg^2+^ supplementation in the prevention and treatment of CVD.

Nevertheless, the aforementioned studies have several limitations. First of all, serum Mg^2+^ does not unequivocally reflect the intracellular concentration of this ion, which better correlates with Mg^2+^ functions [101, 102], thus possibly affecting the results of the studies. Maybe, the evaluation of the intracellular amount of Mg^2+^ within lymphocytes and erythrocytes would be likely more accurate and would correlate better with intramyocardial Mg^2+^ [103]. Secondly, a possible limitation of the cited epidemiological studies is the use of indirect methods, such as food questionnaires, to determine Mg^2+^ intake; thus, it is not possible to discriminate among the effect of Mg^2+^ and residual effect derived from the intake of other microelements. Moreover, it is not possible to determine the amount of supplemented Mg^2+^ effectively absorbed and utilized. Another limitation is the different types of Mg^2+^ formulations used for the supplementation. The studies about the effect of Mg^2+^ intake on CV health use different Mg^2+^ formulations, both organic and inorganic, but it have been demonstrated that there are differences in the bioavailability among formulations. These differences could influence the results of the studies. Lastly, some cited meta-analyses present heterogeneity between the studies, possibly affecting the reliability of statistical analysis. These limitations suggest that new large prospective randomized trial is necessary to elucidate the association among Mg and CV health and to assess the benefits and the usefulness of Mg supplementation in the prevention and treatment of CVD (Figures 2 and 3).

## 4. Conclusions

The present review shows that a relationship between Mg^2+^ and cardiovascular health has been clearly demonstrated and underlines the possible pathophysiological mechanisms through which Mg^2+^ deficiency can promote the onset, progression, and maintenance of CVD. In fact, hypomagnesaemia influences negatively the CV health and is associated with an augmented incidence of hypertension, type 2 diabetes, dyslipidemia, atherosclerosis, arrhythmias, and coronary artery disease. In consideration of the great public-health impact of CVD, the recognition of the detrimental effects of Mg^2+^ deficiency on CV health suggests the possible role of Mg^2+^ blood levels for the screening and prevention of CV risk factors and CVD and helps the identification of new therapeutical strategies for the management of CVD through Mg^2+^ supplementation. This concept is even more shareable if framed in a wider and more complete perspective, that aims to embrace in a global way any possible alteration or disease, even noncardiovascular ones, which can damage the heart health and whose treatment or correction can support specific cardiological therapies, from the standard one to the most innovative, in the pursuit of the healing and the well-being of the patients [104–106]. Considering the limitations of the present studies addressing a role of hypomagnesaemia on the development of CVD, large prospective randomized controlled trials are necessary to elucidate this multifaceted relationship, to assess the benefits of routine Mg^2+^ level assessment in cardiovascular patients and in the general population and the usefulness of Mg^2+^ supplementation in the prevention and treatment of CVD.
